# Structural basis for the participation of the SARS-CoV-2 nucleocapsid protein in the template switch mechanism and genomic RNA reorganization

**DOI:** 10.1016/j.jbc.2024.107834

**Published:** 2024-09-27

**Authors:** Peter R. Bezerra, Fabio C.L. Almeida

**Affiliations:** 1 Program of Structural Biology, Institute of Medical Biochemistry, Federal University of Rio de Janeiro, Rio de Janeiro, Brazil; 2 National Center of Nuclear Magnetic Resonance (CNRMN), CENABIO, Federal University of Rio de Janeiro, Rio de Janeiro, Brazil

**Keywords:** nucleocapsid protein, SARS-CoV-2, coronavirus, RNA-binding, RNA chaperone

## Abstract

The COVID-19 pandemic has resulted in a significant toll of deaths worldwide, exceeding seven million individuals, prompting intensive research efforts aimed at elucidating the molecular mechanisms underlying the pathogenesis of SARS-CoV-2 infection. Despite the rapid development of effective vaccines and therapeutic interventions, COVID-19 remains a threat to humans due to the emergence of novel variants and largely unknown long-term consequences. Among the viral proteins, the nucleocapsid protein (N) stands out as the most conserved and abundant, playing the primary role in nucleocapsid assembly and genome packaging. The N protein is promiscuous for the recognition of RNA, yet it can perform specific functions. Here, we discuss the structural basis of specificity, which is directly linked to its regulatory role. Notably, the RNA chaperone activity of N is central to its multiple roles throughout the viral life cycle. This activity encompasses double-stranded RNA (dsRNA) annealing and melting and facilitates template switching, enabling discontinuous transcription. N also promotes the formation of membrane-less compartments through liquid-liquid phase separation, thereby facilitating the congregation of the replication and transcription complex. Considering the information available regarding the catalytic activities and binding signatures of the N protein–RNA interaction, this review focuses on the regulatory role of the SARS-CoV-2 N protein. We emphasize the participation of the N protein in discontinuous transcription, template switching, and RNA chaperone activity, including double-stranded RNA melting and annealing activities.

To date, the COVID-19 pandemic has killed more than seven million people worldwide, leading to major efforts toward understanding the molecular basis of the disease caused by infection with the virus SARS-CoV-2. This pathogen emerged in 2019, probably following a species crossover from a bat coronavirus. It belongs to the genus Betacoronavirus and comprises one of the largest RNA viruses, having a single-stranded positive-sense RNA genome of 29,903 nucleotides in length. Owing to their large RNA genome, coronaviruses present unique replication and transcription mechanisms. Coronaviruses have RNA proofreading machinery, which is unique to RNA viruses (1, 2).

Despite the rapid development of effective vaccines and drug treatments, COVID-19 continues to be a threat to the human population due to the rapid and continuous emergence of novel variants and the still largely unknown long-term effects of the disease, commonly referred to as long COVID-19. Long COVID-19 affects a significant fraction of the population infected by the virus (∼40%), causing serious health problems and presenting various symptoms, such as fatigue, insomnia, dyspnea, cognitive impairment, and others. The vaccines target the spike protein (S), which is the most immunogenic for neutralizing antibodies (3); it is exposed on the virus surface and is responsible for the recognition of the angiotensin-converting enzyme-2 (ACE2) receptor and integrins that facilitate virus entry (4). Drug development has focused primarily on RNA-dependent RNA polymerase (NSP12), which is responsible for viral transcription and replication (*e.g.*, molnupiravir and remdesivir), and the main SARS-CoV-2 protease NSP5 (*e.g.*, nirmatrelvir and ritonavir) (3).

The nucleocapsid protein (N) is the most conserved and abundant protein produced by coronaviruses. It is essential for the efficient replication and transcription of the viral genome (5) and plays a role in numerous regulatory functions, including its involvement in the discontinuous transcription process, which is unique to coronaviruses and occurs through the formation of subgenomic mRNAs (sgmRNAs) (1).

The virus hijacks and remodels the host cell, equipping it with an efficient apparatus for viral replication and transcription. The virus replication and transcription complex (RTC) primarily involves nonstructural proteins (NSPs) that are compartmentalized within a reticulovesicular network extruded from the endoplasmic reticulum, forming a double-membrane vesicle (DMV), which serves as the RNA synthesis machinery and contains the RTC (6). Recent cryo-electron tomography data revealed the *in situ* structure of a DMV-spanning portal, named the replicopore, which is composed mainly of NSP3 and NSP4, along with other proteins (7, 8). The primary function of the replicopore is to export newly synthesized sgmRNA and viral genomic RNA (gRNA) to the cytoplasm. Double-stranded RNA (dsRNA), which serves as a template for both replication and transcription, can be directly visualized within DMVs (8). N is the first protein expressed and accumulates around the endoplasmic reticulum (ER) (9, 10) and replicopore (8), likely playing a role in this process.

N is a multifunctional protein with a crucial role in packaging gRNA for nucleocapsid assembly (11, 12). This packaging occurs in the cell's secretory pathways, outside the DMVs (13). N also functions as a regulatory protein involved in various aspects of the viral life cycle. Additionally, N plays a role in modulating innate immunity by inducing the expression of proinflammatory cytokines and activating the NLRP3 inflammasome (14), which ultimately contributes to the cytokine storm, one of the key pathogenic mechanisms of SARS-CoV-2. N is also found outside the cell and is robustly expressed on the surface of infected cells, where it regulates the innate immune response by sequestering chemokines (15). At the cell surface, N binds to glycosaminoglycans (GAGs), and GAG-deficient mammalian cell lines have been shown to fail to bind N (15). Recently, the interaction between N and GAGs has been observed *in vitro via* nuclear magnetic resonance (NMR) (16).

Overall, in coronavirus-infected cells, the N protein is found in the cytoplasm outside the DMV, near the endoplasmic reticulum, and surrounding the DMVs (8, 17). N is also found inside the DMV, which is induced by NSP3, NSP4, and NSP6 proteins that produce the membrane curvature necessary for DMV formation (18). DMV morphogenesis and how the N protein enters DMVs remain unresolved. Nevertheless, there is solid experimental evidence that the viral NSPs and the N protein are subunits of the coronavirus RTC and are likely encapsulated inside the DMV during morphogenesis (19). The RTC inside DMVs promotes the synthesis of dsRNA, which serves as a template for replication and sgmRNAs. The DMV membranes protect viral RNA, preparing it for export for subsequent virion assembly and viral protein biosynthesis (8). After viral RNA is synthesized within DMVs, it is exported to the cytoplasm, where it associates with the abundant N protein to form RNPs at the secretory pathways for virus packaging (8). On the cytosolic side, abundant sgmRNAs encounter ribosomes and start the translation. Based on this information, we suggest that the colocalization of the N protein and dsRNA creates conditions conducive to the chaperone activity discussed in this review, primarily inside the DMV.

In SARS-CoV-2, the N protein is never found inside the host cell nucleus, as will be discussed later. Additionally, N is also found in newly formed virions in secretory pathways (13). Moreover, the N protein has been observed in certain subcellular compartments, such as stress granules and processing bodies (P-bodies), which are involved in RNA metabolism and cellular stress responses.

N is the only structural protein present in the RTC (19). It promotes liquid–liquid phase separation (LLPS), fostering the formation of membrane-less compartments, which assist in the assembly of the RTC (20, 21, 22, 23, 24). LLPS possibly aids in the segregation of autologous and heterologous proteins for RTCs (20). N is also described as tailoring the infected cell for the virus by counteracting the formation of nucleic acid stress response organelles, specifically stress granules, which are liquid condensates responsible for cellular antiviral activity. Thus, N plays a role in remodeling the host cell to favor SARS-CoV-2 replication (20, 25, 26, 27). In the case of SARS-CoV-2, viral transcripts dominate the transcriptome of infected host cells, suppressing the expression of host genes (28).

The N protein is largely nonspecific toward RNA recognition, a promiscuity that is important during nucleocapsid assembly. The interaction of the N protein with RNA promotes oligomerization, forming the ribonucleoprotein (RNP) complex (29). RNPs compact positive-sense single-stranded RNA into a helically symmetric structure, which plays a key role in virus assembly. Despite its nonspecificity, N has multiple specific intracellular activities (30), such as recognizing polyadenine tails (poly-A) and regulating the expression of viral structural and accessory proteins by binding to transcription regulatory sequences (TRSs) (1). This is a complex and dynamic process that is still not fully understood. The N protein binds to the poly-A tail with high affinity, inhibiting translation; however, the molecular basis for the specificity of this process remains unknown (31). Another specific activity of N is its role in suppressing the RNA silencing mechanism in mammalian cells (32). The mechanism of RNA interaction, the molecular basis for specificity, and the allosteric effects of the interaction are not well understood.

Considering the available information regarding the structure, dynamics, catalytic activities, and binding signatures of the N protein–RNA interaction, this review focuses on recent advances in understanding the regulatory role of the SARS–CoV–2 N protein. We emphasize the participation of the N protein in discontinuous transcription, the template switch of the nascent negative RNA strand, and its RNA chaperone activities, including double-stranded RNA (dsRNA) melting and annealing. An essential review focusing on the role of N in the template switch mechanism was published by Sola *et al.* (1). Several other recent reviews highlight the importance of N in SARS-CoV-2 pathogenicity: immunogenicity and next-generation vaccines (14, 33, 34, 35); immune evasion and inflammation (36, 37); structure, disorder, and pathogenesis (38, 39, 40); the role of nucleocapsid proteins in LLPS (41, 42); the use of the N protein for laboratory diagnosis (43); the use of N as a potential antiviral target (44); and the multifunctional aspects of the N protein (37, 45).

### Nucleocapsid protein structure

The N protein from betacoronaviruses contains two independent globular regions, the N-terminal (NTD) and the C-terminal (CTD) domains (46), along with three intrinsically disordered regions (IDRs: N-arm, linker, and C-tail, Fig. 1*A*). The NTD and CTD are interconnected by a linker that includes a serine-arginine-rich motif (SR, ^177^RGGSQASSRSSSRSRNSSRNSTPGSSR^203^) (29, 45, 47). These structural features are conserved among betacoronaviruses and enable a wide range of interactions with various partners. Numerous studies have demonstrated interactions between the N protein and nucleic acids across every domain *via* different and complementary methodologies, such as NMR (21, 47, 48, 49), paramagnetic relaxation enhancement (50), X-ray crystallography (51, 52), electron microscopy (53), and optical tweezers (54).

**Figure 1:**
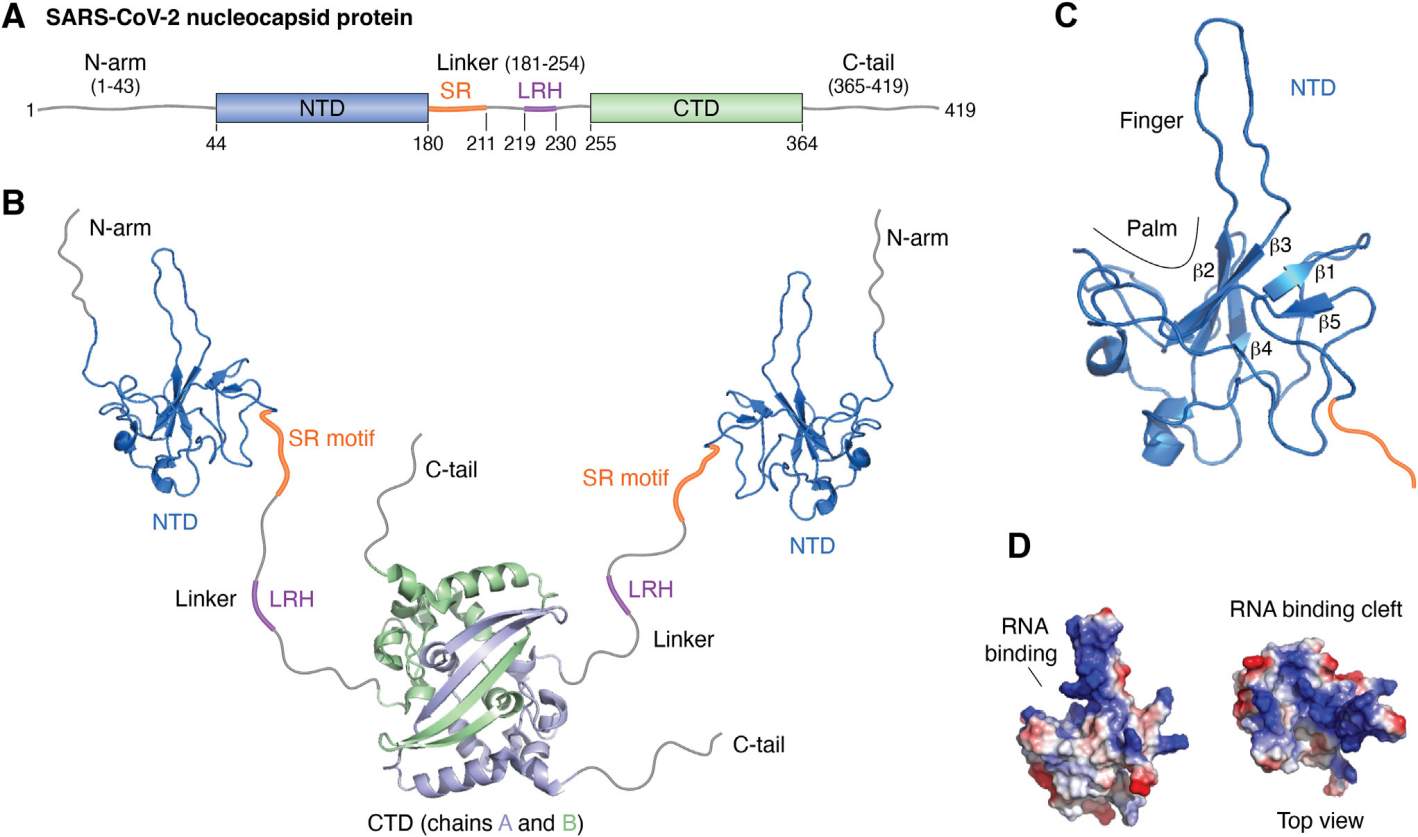
**SARS-CoV-2 nucleocapsid protein structural organization.***A*, schematic representation of the primary sequence distinguishing all structural regions. The rectangles represent the globular domains, the N-terminal (NTD, 44--180) and C-terminal (CTD, 255--364) domains. The *gray lines* represent the three intrinsically disordered regions: the N-arm (1--43), linker (181--254), and C-tail (365--419). The *orange line* shows the serine-arginine-rich motif (SR motif) (181--211) and the *purple line* shows the leucine-rich helix (LRH) (219--230) within the linker IDR (181--254). *B*, representation of the structural elements of the N protein. The structure of the globular domains was obtained from the following PDB identifiers: NTD, 6YI3, CTD, and 7VBF. The NTD (*blue*) is the regulatory RNA-binding domain, whereas the CTD (*light blue*, chain A, and *light green*, chain B) is the dimerization domain, which also binds RNA, mainly in the nucleocapsid assembly. The *gray lines* represent the IDRs, the *orange lines* represent the SR motif, and the *purple lines* represent LRH. The orientation of the IDRs and NTD relative to the CTD domain was freely sketched to reflect the dynamic behavior due to the IDRs. *C*, NMR structure of the NTD highlighting the binding cleft formed by the finger and palm (6YI3). The finger is composed of a β2 to β3 loop, and the palm is composed of the surface of the twisted β-sheet (β1–β5). Note that the SR region (*orange*) is immediately adjacent to the C-terminus of the NTD. *D*, the electrostatic surface potential of the NTD showing the positively charged RNA binding cleft (*blue*) located *right* between the palm and the finger. The negatively charged potential is shown in *red*, and the neutral potential is shown in *white*. The structures were generated with PyMOL version 2.5.4 Schrödinger, LLC (112).

Both the NTD and CTD are RNA-binding domains (RBDs). The NTD binds RNA with sequence specificity (21, 48, 50, 55), whereas the CTD is mostly nonspecific. The N-arm and linker regions are essential for modulating RNA binding affinities (56). The SR region is crucial for the melting activity of the NTD (21, 55). The linker region promotes the formation of a dynamic complex with NSP3 through a leucine-rich hydrophobic helix (LRH, ^219^LALLLLDRLNQL^230^) and a polar disordered region (^243^GQTVTKKSAAEAS^255^), a protein–protein interaction essential for targeting N to the replication and transcription complex (RTC) (49).

The CTD serves as the dimerization domain (Fig. 1*B*) (30), and the C-tail mediates interaction with the membrane protein (M) in the assembled virus particle. The CTD is pivotal for RNP assembly (57). Nucleic acid-bound N-protein dimers oligomerize *via* a protein–protein interface presented by the transient LRH in the linker between the NTD and CTD. The nucleocapsid assembly is then stabilized by the dimeric CTD (58). RNP-like assemblies can be obtained *in vitro via* small oligonucleotides (59), indicating that the assembly depends primarily on the protein and less on specific interactions with the genomic RNA. A truncated N protein lacking the N-arm, NTD, and SR region (Δ1--209) can also assemble into an RNP-like particle *in vitro* (59).

Phosphorylation plays a key role in regulating the multiple functions of N. The SR region is hyperphosphorylated in the early stages of infection and likely becomes phosphorylated when the first copies of N are expressed and encapsulated at the DMV as a subunit of the RTC. This remains an outstanding question that requires further study. RNP assembly is downregulated by phosphorylation of the N protein in the SR region, which is located within the linker between the two globular domains (29). Conversely, phosphorylation is required for the regulatory functions of N, influencing its localization and participation in the RTC. Phosphorylation also modulates the properties of the liquid condensates formed by the N protein. Unphosphorylated N forms a gel-like phase, whereas the phosphorylated protein induces more liquid-like dynamic condensates (24). The inhibition of glycogen synthase kinase 3 (GSK-3) prevents N phosphorylation and impairs SARS-CoV-2 transcription and replication (60). GSK-3 inhibition also decreases the generation of genomic and long subgenomic mRNAs (sgmRNAs) in cells infected with SARS-CoV (61).

The N protein's RNA chaperone activity is primarily related to transcription and replication within the DMVs (10), promoting the melting and annealing of double-stranded RNA and gRNA (10, 21, 48, 55, 62). It is unlikely that the N chaperone activity plays a role in RNP assembly. It has been reported that the N protein of SARS-CoV is dephosphorylated/hypophosphorylated within the virion (58, 63). These phosphatase activities are still poorly understood, but there are reports showing that the M protein interacts with various host cell phosphatases (29, 64).

The SR region is hyperphosphorylated in infected cells, making it important to understand the role of GSK-3 in phosphorylating multiple serine residues (12 putative sites) in the SR region. The mechanism that drives the switch from the assembly-prone unphosphorylated state to the regulatory-prone state is still not fully understood. The assembly depends on N protein for protein–RNA and protein–protein interactions. Phosphorylation weakens these interactions resulting in less compact RNPs (65). NMR data show that the unphosphorylated state tends to dimerize or aggregate through LRH, and hyperphosphorylation weakens these associations (66). The authors demonstrated that the unphosphorylated full-length N undergoes a dimer/tetramer equilibrium, which shifts toward tetramers in the presence of RNA. Monophosphorylation at S188 allows RNA to bind to the NTD but not to the SR, reducing protein compaction. Hyperphosphorylation leads to the dissociation of the four-helix bundle of the LRH, ultimately stabilizing the dimeric conformation, which is more conducive to regulatory functions. In the hyperphosphorylated dimer, RNA binds mainly to the NTD. In addition to (hyper)phosphorylation of the SR region, the N protein undergoes other posttranslational modifications, such as glycosylation, methylation, and acetylation, which are involved in other regulatory functions (39).

To our knowledge, there is only one structural model describing how the full-length N protein interacts with RNA. Ribeiro-Filho *et al.* (53) demonstrated through electron microscopy that, in the absence of RNA, the full-length N dimer exhibits significant dynamic flexibility in the N-arm, NTD, and linker regions. This dynamic flexibility is likely crucial for the regulatory functions of the N protein. In the presence of RNA, N undergoes domain compaction, with the RNA interacting with all the protein domains. The group also reported a supramolecular association of N, which formed ring-like octamers in the presence of 50-nucleotide-long poly-A RNA. It is not yet known whether this oligomeric form plays a role in RNP assembly.

The specific function of N is attributed to the NTD (21). The NTD structure resembles a hand-like fold. The “palm” is composed of a twisted β-sheet formed by β1/β2/β3/β4/β5, and the “finger” is formed by a long loop that projects between β2 and β3 (Fig. 1*C*). The palm and finger create the nucleic acid binding cleft, which features a positively charged electrostatic surface (Fig. 1*D*). This characteristic is conserved among coronaviruses (29, 46).

Important structural information about IDRs has come from NMR studies, which provide site-specific insights through the chemical shifts of each nucleus. These chemical shifts reveal the local chemical microenvironment, which depends on the local structure and dynamics. The nearly complete NMR resonance assignment of a construct containing the N-arm, NTD, and linker (67) has enabled the description of specific sequence signatures involving the IDRs of the N protein. Upon titration of this construct (N-arm, NTD, and linker) with RNA, the SR region was perturbed in the early stages (56). The linker initiates and enhances the binding of N to RNA (56). Electrostatic is the primary force driving RNA recognition by IDRs. An arginine-rich region of the N-arm also plays an important role in RNA binding (^32^RSGARSKQRRPQGLP^46^). The LRH region (^216^DAALALLLLD^225^) was perturbed upon RNA binding, resulting in chemical shifts and intensity changes. Intensity changes indicate alterations in protein dynamics. This region is the same that binds NSP3 (49), and the LRH region is involved in intramolecular interactions with other regions of the protein that are perturbed in the presence of RNA (56).

Multiple viral proteins possess nuclear localization signals (NLSs) and nuclear export signals (NESs), enabling them to shuttle between the cytoplasm and the nucleus. The accumulation of viral proteins in the nucleus can reduce viral infection (68), and nuclear transport is required for successful viral assembly in the cytoplasm (69). The N protein of many viruses within the order *Nidovirales* has been reported to localize in both the cytoplasm and the nucleus, specifically at the nucleolus (70). The function of the N protein in the nucleolus is associated with the regulation of subgenomic RNA (sgRNA) synthesis and its association with ribosomal subunits (30). Coronavirus N proteins have conserved NLS and NES sequences (69, 70, 71). While their nuclear targeting mechanism has not been fully described, hypotheses include both active and passive transport through the nuclear pore complex. The N proteins of SARS-CoV-2 and SARS-CoV have been reported to localize exclusively in the cytoplasm, despite the presence of NLSs (72, 73). It is not yet clear whether the N protein does not enter the nucleus or if it remains in the cytoplasm because the balance between nuclear import and export is skewed toward export.

The LRH located in the linker region acts as an NES. Truncated constructs of the SARS-CoV N protein lacking the LRH have been reported to localize in the nucleolus (71), indicating that the nuclear export rate is regulated by the presence of this motif. Interestingly, differences in the accessibility of LRH could explain the variation in the subcellular localization of the N protein among different *Nidovirales* (30). Importantly, the NES motif is involved in internal hydrophobic interactions (56), suggesting that nuclear localization may be regulated by the conformation and dynamics of the N protein.

## Discontinuous transcription and template switching

In this section, we review the template switch mechanism before describing the participation of the N protein in facilitating this process. Transcription and replication in viruses of the order *Nidovirales* use different mechanisms. While replication is a continuous process, transcription is discontinuous. Discontinuous transcription allows for the expression of different proteins at varying levels, making it an important regulatory mechanism (Table 1). In discontinuous transcription, a segment of the template RNA is skipped to create a nested sgRNA. This differs from other positive-sense RNA viruses, such as *Flaviviruses*, which transcribe and translate their genome into a single polyprotein that is subsequently processed by proteases into the individual structural and nonstructural proteins of *Flaviviruses*. As a result, *flaviviruses* express all proteins in equal amounts. Discontinuous transcription also occurs in other RNA viruses but is not the predominant transcription mode. In coronaviruses, discontinuous transcription is a regular mode of transcription. It is highly specific and efficient, making this mechanism the common route for transcription (1, 28, 74). This process enables the differential expression of structural and accessory proteins, contributing to the evolutionary adaptation of viruses.

**Table 1:** SARS-CoV-2 TRS-dependent subgenomic messenger RNA (sgmRNA) levels during infection

sgmRNA	Normalized count (ARTIC nanopore)	Normalized count (ARTIC illumina)	Normalized count (dRNAseq)
TRS - S	937.06	466	1336.5
TRS – ORF3a	304.77	213.58	8961.39
TRS - E	109.17	139.8	2989.98
TRS - M	1871.85	454.35	25,504.83
TRS – ORF6	441.24	396.1	5551.61
TRS – ORF7a	257.01	54.37	20,133.14
TRS – ORF7b	9.1	–	19.94
TRS – ORF8	186.5	128.15	5371.69
TRS - N	2858.94	2438.75	64,169.02
TRS-dependent noncanonical	74.96	81.83	1576.28

Normalized counts of TRS-dependent sgmRNAs in reads per million (RPM = read count/total number of reads mapped to the reference genome ∗ 1,000,000) of hACE2-A549 cells infected with SARS-CoV-2. Sequenced data were obtained through nanopore direct RNA sequencing (dRNAseq) and an amplicon-based approach (ARTIC) *via* nanopore or illumina (76).

**Figure 2:**
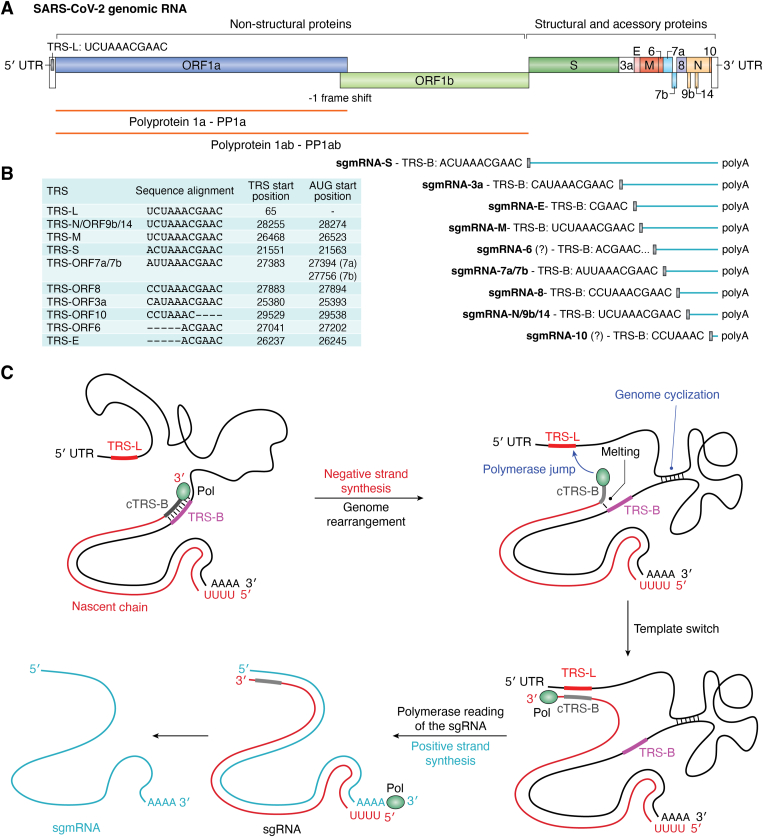
**Template switching, discontinuous transcription, and transcription regulatory sequences of SARS-CoV-2.***A*, representation of the genomic RNA (gRNA) of SARS-CoV-2, highlighting the open reading frames (ORFs) for the nonstructural protein and structural and accessory proteins. The structural and accessory regions contain TRS-B upstream of the ORF (*black rectangles*). The polyproteins pp1a and pp1ab are represented in *orange*. The *blue lines* represent the sgmRNAs for each ORF of the structural and accessory proteins. We show the TRS-B sequences for each ORF. The question mark (?) at TRS-ORF6 is due to the high distance between the TRS-B (27041) and AUG (27202), and for TRS-ORF10 is because the sgmRNA has never been detected, despite the presence of TRS-B. *B*, alignment of the TRSs of SARS-CoV-2, showing the TRS start position and the start codon (AUG) position in the gRNA. *C*, schematic mechanism of the canonical TRS-dependent template switch: first, the nascent negative-sense chain (*red*) is encoded by the RNA-dependent RNA polymerase, represented as a *green ellipse*. In addition to negative-sense synthesis, genome rearrangement prompts the genome to adopt a conformation suitable for polymerase jumping, favoring the template switch. Once cTRS-B is synthesized, the negative-sense strand detaches through the melting of dsTRS-B. The template switch occurs through the RNA polymerase jump, promoting base pairing (annealing) between TRS-L and cTRS-B. The cyclization of the gRNA placing the cTRS-B close to the TRS-L is essential. After cTRS-B/TRS-L annealing, the polymerase continues to encode until the 5′ end of the gRNA. The resulting nascent chain is the sgRNA (*red*), which is used as a template for the RNA polymerase to synthesize the sgmRNA (*light blue*).

Transcription in *Nidovirales* occurs through two different mechanisms. The transcription of ORF1a and ORF1b is continuous, resulting in the translation of two polyproteins: PP1a and PP1b (Fig. 2*A*). The polyprotein PP1b is generated through a −1 base frameshift caused by a pseudoknot RNA structural frameshift element located between ORF1a and ORF1b (75). These polyproteins are subsequently processed by proteases to produce nonstructural proteins.

The transcription mechanism for structural and accessory proteins occurs discontinuously through the formation of TRS-dependent sgmRNAs (Fig. 2*A*), including the body TRS (TRS-B), which encodes ORF10. This discontinuous transcription mechanism involves TRS sequences located at the 5′ ends of each of the 9 ORFs (TRS-B) and at the 5′ untranslated region (5′UTR) (leader TRS–TRS-L). These TRS sequences are conserved gRNA motifs (Fig. 2*B*) that mediate the template switch mechanism (Fig. 2*C*). The complementarity between each TRS-B of the negative-sense nascent chain (cTRS-B) and the positive-sense TRS-L facilitates the jump of RNA polymerase from the 5′ ends of each ORF to the 5′UTR, promoting the formation of shorter negative-sense sgRNAs (Fig. 2*C*). The RNA polymerase subsequently transcribes the sgmRNA using the sgRNA as a template. The efficiency of the template switch mechanism depends not only on TRS-L/cTRS-B complementarity but also on the rearrangement of the genome into a conformation suitable for the transcription of each sgRNA. This process involves genomic cyclization, which will be further discussed in relation to the different genome architectures described for SARS-CoV-2.

The number of assigned TRS-dependent canonical sgmRNAs is reported to be nine in two of the most important recent studies of the transcriptome of SARS-CoV-2-infected cells (28, 76), excluding ORF10. These studies did not identify a sgmRNA for ORF10, despite the presence of TRS-B (Fig. 2*A*). The authors also presented transcriptome data showing two different sgmRNAs for ORF7a and ORF7b, despite the absence of TRS-B for ORF7b, which is reported as a single nucleotide, A (76). Here, as shown in Figure 2*A*, we propose the possibility of a single TRS-B assignment for both ORF7a and ORF7b, which would produce a single sgmRNA containing both ORFs. This interpretation remains open for discussion and future experimental studies.

Notably, most transcriptome studies of SARS-CoV-2 have considered the TRS core sequence (CS) as only ACGAAC, which is present in all TRSs except for ORF10 and ORF7b (Fig. 2*B*). In this review, we propose an extended version of the TRS CS, including the preceding five nucleotides, where the UAAAC sequence is conserved in eight TRSs in SARS-CoV-2 and SARS-CoV, including the TRS-L (Fig. 2*B*). A nucleotide BLAST (BLASTN) search using the SARS-CoV-2 TRS-L sequence (UCUAAACGAAC) as the query revealed that many betacoronaviruses closely related to SARS-CoV-2 have TRS sequences lacking ACGAAC and present only the UAAAC sequence as a conserved core. We believe that this observation justifies considering the UAAAC sequence as an extension of the TRS. Examples include bat coronaviruses (NC_014470.1, NC_025217.1), *Rattus norvegicus* betacoronavirus HKU24 (NC_026011.1), human betacoronaviruses HKU1 and OC43 (NC_006577.2 and NC_006213.1), rabbit betacoronavirus HKU14 (NC_017083.1), MHV betacoronavirus (NC_001846.1 and NC_048217.1), and bovine betacoronavirus (NC_003045.1), among others. The UAAAC sequence contains the recognition motif for N protein melting activity, as discussed later in the review.

Historically, discontinuous transcription was first identified by Spaan *et al.* through the characterization of mRNAs in cells infected with the murine hepatitis virus betacoronavirus (MHV) (77). The authors obtained cDNAs from these mRNAs and annealed them with the viral gRNA. Electron micrographs of the hybrid cDNA/gRNA revealed the presence of a common leader sequence in two different MHV mRNAs. They proposed a polymerase jump mechanism to explain the synthesis of these short RNA transcripts from the 3′ end of the negative-stranded template (illustrated in Fig. 2*C*). In 1987, Baric *et al.* (78) identified multiple leader-containing short mRNAs. Using leader-specific cDNA probes, they detected small RNAs in MHV-infected cells and demonstrated that these were functional transcripts encoded by the MHV gRNA. They proposed a model for discontinuous transcription mediated by leader RNA containing the 5′-UCUAAA-3′ core sequence, which was later designated TRS-L.

In 2004, Zúñiga *et al.* (79) proposed a three-step model to explain discontinuous transcription: (i) protein-mediated gRNA 5′/3′ cyclization (genome rearrangement, Fig. 2*C*), (ii) base pairing scanning of the nascent negative-sense RNA by TRS-L, and (iii) synthesis of the negative-sense sgRNA through a template switch mechanism (Fig. 2*C*). One important observation by the authors was that mutations in the TRS core sequence (CS) did not abolish but did decrease the transcription level of the sgRNA by up to 10³-fold. These data were obtained *via* the quantification of mutated CS-B RNAs *via* real-time PCR (RT–PCR) with specific oligonucleotides. This observation highlights the importance of TRS sequence specificity in priming the synthesis of negative-sense sgRNA. This finding suggests that the template switch depends largely on a suitable conformation of the gRNA that positions the complementary TRS-B close to TRS-L, facilitating polymerase jumping and consequently the template switch. In agreement with these findings, TRS-independent template switching has recently been described for SARS-CoV-2 transcription as a rarer event (∼7%) (28). TRS-dependent canonical and noncanonical discontinuous transcription accounts for ∼93% of the sgRNAs produced during SARS-CoV-2 infection (28). Canonical TRS-dependent template switching constituted 92.6% of the detected sgmRNAs in SARS-CoV-2 (28). To reach this conclusion, the authors mapped the transcriptome and epitranscriptome of SARS-CoV-2-infected cells. Both TRS-independent (7%) and TRS-dependent noncanonical template switches (0.4%) contribute to the generation of imperfect homologous recombination, which impacts coronavirus evolution. Chrisman *et al.* (80) analyzed SARS-CoV-2 genome sequences from GISAID and cataloged over 100 insertions and deletions. They reported that these insertions and deletions are more frequent at template-switch hotspots during the evolution of SARS-CoV-2, suggesting that template-switching plays a role in the genetic variability of the virus. For a review of the evolution of SARS-CoV-2, see Markov *et al.* (81).

An important contribution to understanding the gRNA conformational demands for the template switch was made for transmissible gastroenteritis virus (TGEV). TGEV is classified in the genus *Alphacoronavirus*, subgenus *Tegacovirus*, and species *Alphacoronavirus 1*. Mateos-Gomez and colleagues identified specific genomic regions of TGEV involved in RNA secondary structures that mediate long-range interactions. The authors engineered TGEV replicons to investigate the requirements for long-distance genome interactions and the complementarity of RNA sequences (82). A hairpin located between nucleotides 26,356 and 26,438, referred to as the active domain (AD), exhibits long-range complementarity (nucleotides 26,412–26,421, B) to a region downstream of the 5′-UTR, between nucleotides 477 and 486 (cB). This long-range base pairing facilitates a rearrangement of approximately 26,000 nucleotides, bringing the TRS-L into proximity with the TRS-B of the ORF encoding the N protein (82).

In the case of the N ORF of TGEV strains, another secondary structure element exists between a proximal element (pE), situated seven nucleotides upstream of the TRS-B of the N protein, and a distal element (dE), located 449 nucleotides upstream of TRS-B N. Both pE and dE are highly conserved among alphacoronaviruses. The authors reported that the distance between pE and dE (*i.e.*, the size of the loop) is inversely proportional to the amount of transcribed sgmRNA of the N protein in TGEV-infected cells. They hypothesized that these long-range interactions guide the polymerase's action (83, 84). The maintenance of secondary structures and specific sequences within the gRNA appears to be essential for the efficient synthesis of sgmRNAs. High-order RNA–RNA interactions, along with genome structure rearrangements, are required to facilitate discontinuous transcription in Nidovirales (82). However, the mechanisms observed for TGEV are not necessarily conserved in betacoronaviruses. Important new information on the conformational arrangements of SARS-CoV-2 gRNAs will be discussed later in this review.

This contribution to the understanding of the TGEV template switch mechanism highlights the critical role of gRNA conformational changes in regulating polymerase jumping, which, in turn, influences RNA recombination events. Furthermore, the direct involvement of the N protein likely enhances the efficiency of the template switch process.

### Specific roles of the N protein in the template switch

The findings described above highlight the critical role of TRSs in discontinuous transcription. As mentioned earlier, the template switch relies primarily on the structural dynamics of gRNA (interconversion among different genome architectures) and can occur independently of the TRS, albeit at a low frequency. The presence of TRSs, which facilitate the complementarity between TRS-L and cTRS-B, significantly enhances the efficiency of this process. One of the key factors in this “catalysis” promoted by TRS-dependent recombination is the specific recognition of TRSs by the N protein. In the following section, we present experimental evidence demonstrating how the N protein enhances replication and transcription, particularly discontinuous transcription through the TRS-dependent template switch. The RNA chaperone activities of the N protein, which involve promoting the melting or annealing of duplex RNA and hairpins, are central to this role.

Baric *et al.* reported that sgmRNAs could be immunoprecipitated from lysates of murine hepatitis virus (MHV)-infected cells *via* the use of anti-N monoclonal antibodies (85). They demonstrated that the MHV N protein specifically recognizes the TRS present in all sgmRNAs. In a related study, the same group identified the location of TRS-L in the gRNA, spanning bases 56 to 72 (5′-UUAAAUCUAAUCUAAAC-3′) of MHV. Through competition assays, they showed that the N protein binds to the TRS-L site with high affinity (∼14 nM) (86, 87). Additionally, Compton *et al.* reported that anti-N antibodies inhibited the *in vitro* replication of MHVs (88). They used extracts from MHV-infected cells and measured the incorporation of radiolabeled ^32^P-UMP into genome-sized RNA. The addition of anti-N antiserum inhibited gRNA synthesis by 90%, indicating that the N protein directly impacts the efficiency of gRNA replication. These early observations of N protein binding to gRNA are supported by recent RNA interactome studies, which show that the N protein is by far the most abundant protein that binds to SARS-CoV-2 gRNA (89, 90). Recent transcriptome analyses, which examined TRS-dependent sgmRNA transcription levels *via* three different RNA sequencing methods, also identified N as the most abundantly expressed protein in infected cells (Table 1) (76).

The essential role of the N protein in viral replication was further demonstrated in 2009 by Grossoehme *et al.* (55). They reported that a single point mutation (Y127A in MHV, corresponding to Y109 in SARS-CoV-2) within the N-terminal domain (NTD) nearly abolished virus infectivity. *In vitro* binding assays revealed that TRS-specific RNA recognition and its regulatory function are inherent to the NTD. The MHV NTD can linearize stem–loop 3 (SL3), which contains the TRS-L core sequence (CS), and melt the double-stranded TRS-L. This study also highlighted the significant specificity of the N protein for the triple-A motif (^68^AAA^70^). More recent NMR studies have confirmed that the NTD is the primary site for RNA binding and that multivalency leads to tighter binding of the full-length N protein to the first 1000 nucleotides of the SARS-CoV-2 gRNA (91, 92). The highly conserved Y127 is centrally located within the NTD's RNA-binding cleft (palm). The motif ^124^PRWYFYYLGTGP^135^ (^106^PRWYFYYLGTGP^117^ in SARS-CoV-2), which forms the β3 strand, is widely conserved among betacoronaviruses. Royster *et al.* further illustrated the potential of N as a target for drug discovery. They described how the inhibition of SARS-CoV-2 N by a novel aromatic compound (K31) significantly reduced virus infectivity (93).

Although the evidence highlights the critical role of the N protein in viral replication and transcription, its multifunctional nature makes it challenging to isolate its specific contributions to each of the virus mechanisms. The significant impact of N on viral infectivity (55, 93) underscores its importance but does not clearly delineate which of its many functions, whether in transcription, replication, packaging, liquid–liquid phase separation (LLPS), or other processes, has the most profound effect. Despite this complexity, there is direct evidence of N involvement in *in vitro* gRNA replication (88) and strong indirect evidence of its role in discontinuous transcription, particularly in the template switch mechanism. This is supported by N's direct binding to sgmRNA in infected cells (85) and its specific, high-affinity recognition of the TRS core sequence (21, 55, 86). Further discussion in this review will demonstrate that N's ability to melt and anneal the double-stranded TRS (dsTRS) provides additional evidence of its crucial role in the template-switching mechanism.

**Figure 3:**
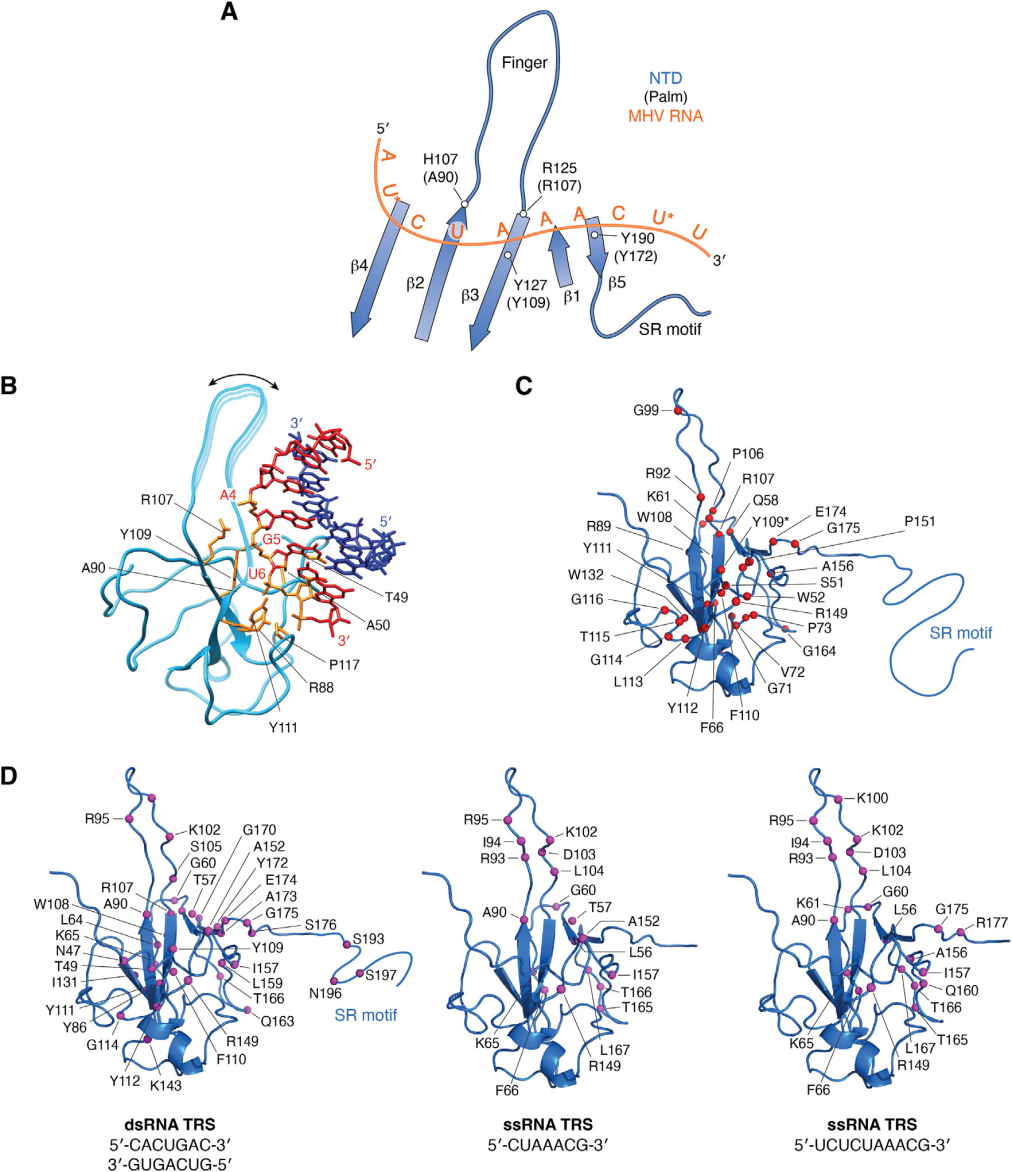
**RNA recognition by the NTD.***A*, schematic representation of the palm of the NTD (β1–β5) showing the interacting MHV RNA strand (TRS, AUCUAAACUU) in *orange*. The binding polarity was determined by paramagnetic resonance relaxation enhancement (PRE) (50). The TRS-L binding orientation is defined by the proximity to the nitroxide spin label at U2 and U9 (*asterisk*). Residues H107, R125, Y127, and Y190 stand out as important for the interaction. The homologous residues in the SARS-CoV-2 NTD are shown in parentheses: A90, R107, Y109, and Y127. *B*, ribbon representation of one of the poses of the crystal structure (PDB ID: 7XWZ) (52) of the complex between the SARS-CoV-2 NTD and a dsRNA (5′-GUCAGUG-3’ (*red*) and complementary strand (*blue*)). The residues participating in the interaction are in *orange*. The finger region is not observed in the structure because of its flexibility. We drew the finger in *cyan* to represent its dynamics, possibly transiently embracing the dsRNA. *C*, SARS-CoV-2 N-NTD structure (PDB ID: 6YI3) (*blue*), highlighting the highly conserved residues among betacoronaviruses. Y109 is changed to a phenylalanine in the bat coronavirus HKU4 (*asterisk*). *D*, structure of the SARS-CoV-2 NTD (PDB ID: 6YI3) showing the residues with the largest chemical shift perturbation (CSP) according to Dinesh *et al.* (47).

Despite its importance, many questions remain about the mechanism of N protein RNA recognition. The dynamics of the NTD significantly influence its interaction with RNA, with the plasticity of the NTD playing a central role in the regulatory function of the N protein as an RNA chaperone, facilitating the melting or annealing of dsRNA (21), as will be further discussed in this review. In the NTD, RNA binds to a highly positively charged cleft between the finger and palm regions (Fig. 1*C*). Keane *et al.* identified the polarity of RNA binding to the NTD as one of the key features of this interaction, which they determined *via* NMR paramagnetic relaxation enhancement (PRE). They used spin-labeled single-stranded TRS (ssTRS) of murine hepatitis virus (MHV) (5′-AUCUAAACUU-3′) to explore this interaction (50). PRE is a crucial method in structural biology for measuring transient interactions and involves the labeling of a ligand with a paramagnetic center, such as a stable free radical. In this case, a nitroxide was inserted at the nucleotide residues U2 and U9 of the ssTRS (Fig. 3*A*). The proximity of the paramagnetic center to the protein enhances nuclear relaxation, allowing for the mapping of the interaction site, as demonstrated by the interaction of the MHV ssTRS with the NTD (Fig. 3*A*). For single-stranded RNA, the 5′-end binds deep into the palm, near the β4 strand. Without a high-resolution structure of the complex, the study revealed that residues H107 (β2), R125 (β3), Y127 (β3), and Y190 (β5) in the MHV NTD anchor the triple-A motif (AAA) in the ssTRS RNA. These residues correspond to A90, R107, Y109, and Y172 in the SARS-CoV-2 NTD.

The only high-resolution crystal structure available for an RNA/NTD complex (SARS-CoV-2) reflects the dynamic nature of the interaction (52). The asymmetric unit in the crystal structure is dimeric and reveals two different binding modes for each subunit. The interactions are governed by hydrogen bonds and electrostatic interactions. In both poses, the finger region is not resolved, indicating the dynamics of the interaction with the finger transiently embracing the duplex RNA (Fig. 3*B*). In one pose, the interaction is more superficial, involving R92 and R107 interacting with the phosphates. In the other pose, the dsRNA interacts more deeply in the binding cleft (Fig. 3, *A* and *B*). The interaction polarity is the same as that of the single-stranded RNA described by Keane *et al.* (Fig. 3*A*), with residues A90 (β2), R107 (β3), Y109 (β3), and Y111 (β3) playing a central role in RNA recognition. The authors used a nonspecific RNA sequence, so no information on specificity is provided. Nevertheless, this structure is informative regarding the participation of the β3 triad (R107, Y109, and Y111) in the interaction with the RNA motif 4-AGU-6. R107 forms a salt bridge with the A4 phosphate, whereas Y109 and Y111 form hydrogen bonds with G5 and U6, respectively. In line with the PRE (50) and crystal structure of the NTD/dsRNA complex (52), the crystal structure of the OC43 NTD complexed with GMP shows binding at the β3 triad, corresponding to positions R107, Y109, and Y111 of the SARS-CoV-2 NTD (51). Notably, the conserved residues in the NTD correlate well with the RNA binding cleft described by PRE and X-ray crystallography. Among the conserved residues are positively charged residues responsible for electrostatic interactions, and polar and aromatic residues, such as Y109 and Y111, are likely involved in interactions associated with specificity.

Studies in solution have highlighted the dynamic nature of the NTD/RNA interaction. One of the most informative tools used to study this interaction is chemical shift perturbation (CSP) analysis, which reveals the effects of interactions with single- and double-stranded RNA (Fig. 3*D*). CSP data for the NTD consistently show the involvement of residues in the palm (β1–β5) and the finger (21, 47, 48, 91), with minimal CSP signatures that would allow differentiation between interactions with specific, nonspecific, single-stranded, or duplex RNAs. Additionally, CSP data have revealed a secondary binding site composed of residues L64, K65, F66, G71, Q163, T165, T166, and L167 (21, 91). These features suggest a somewhat promiscuous and dynamic interaction. The remaining question is how a protein with such promiscuous binding characteristics can perform specific functions. Although there is no definitive answer yet, some experimental insights provide valuable clues.

The association of the NTD with nucleic acids is highly dependent on salt and phosphate concentrations, primarily due to the significant role of electrostatic interactions between RNA phosphates and the positively charged residues of the NTD, which are largely nonspecific. *In vitro* fluorescence binding assays have shown that the binding affinities of nucleic acids to NTDs and NTD-SRs range from nanomolar at low salt or phosphate concentrations to micromolar in environments resembling cellular conditions, such as phosphate-buffered saline (PBS) (21). Most of the apparent affinities measured for RNA are in the micromolar range, making distinguishing between specific and nonspecific sequences difficult. However, measuring CSP or peak intensity changes at high ionic strength is one of the most effective methods for identifying specific features of the interaction. Specific interactions between proteins and RNA have been reported to persist even at high salt concentrations (∼400 mM NaCl), resulting in salt-persistent CSP and intensity changes (48). As mentioned earlier, chemical shift perturbations reveal the local chemical environment of specific nuclei, whereas local dynamics are accessed through line broadening, which typically results in decreased peak intensity due to micro-to-millisecond timescale fluctuations. The persistence of CSP and peak intensity changes under high-salt conditions suggests that nucleic acid binding is not solely electrostatic but likely involves hydrophobic contacts that contribute to specificity.

Korn *et al.* (48) used NMR and other biophysical methods to systematically analyze how the NTD binds to RNA secondary structure elements within the 5′ and 3′ regulatory untranslated regions (5′ UTRs and 3′ UTRs, respectively) of the gRNA. They reported a strong preference for the single-stranded 5′ UTR, particularly for the TRS CS-containing SL3, which supports previous findings on the importance of this interaction for the template switch (55, 86, 87).

Additionally, the NTD was found to bind to SL5 and the small secondary structure element extension of SL4 (Ext). Interestingly, the interaction with SL4 alone was not salt persistent, unlike the interaction with either SL4_Ext or Ext alone, indicating that Ext is involved in specific interactions. Experimental probing of the RNA secondary structure revealed that SL4_Ext does not fold into a secondary structure within the full-length gRNA, indicating that this folding is context-dependent (94, 95). These findings align with crosslinking measurements between N and the gRNA, which revealed that N preferentially binds to specific regions characterized by single-stranded RNA flanked by structured elements of the gRNA (96). The authors demonstrated that certain gRNA sequence-specific patterns can promote LLPS, whereas other regions act as inhibitors. The formation of liquid condensates was found to be RNA sequence- and structure-specific, as shown by experiments where oligonucleotides containing the TRS CS were sufficient to form liquid condensates *in vitro*, whereas nonspecific sequences led to solid condensates.

Since the pandemic, several studies have highlighted the specific role of the N protein in promoting LLPS, which results in the formation of liquid condensates that locally regulate the congregation of RTC proteins and, consequently, their local concentration (20, 21, 22, 23, 24). Interestingly, the melting and annealing activities of the N protein are concentration-dependent. At low concentrations, the SARS-CoV-2 N protein exhibits strong annealing activity, whereas at high concentrations, it displays melting activity (62). Further research is needed to fully understand this concentration-dependent behavior and its impact on viral replication and translation. However, these findings suggest that the regulatory function of N may involve both template switching and phase separation, with potential interdependence between these two seemingly distinct functions.

Overall, we still do not have an answer to the question of how a promiscuous protein performs specific functions. There is still not enough information in the literature to fully understand this mechanism. However, the salt dependence of the binding affinities suggests that the local environment of the gRNA strongly influences these interactions. We speculate that the binding affinity to the gRNA must be highly dependent on the gRNA conformation, which may regulate the exposure of specific structural elements and the local ionic concentration by limiting the access of ions to the binding site. This speculation is supported by seminal experiments reporting a nanomolar affinity (∼14 nM) for the binding of N with the TRS in the gRNA of MHV (86, 87), which contrasts with the micromolar affinities measured for small RNA structural elements. Further in this review, we describe what is known about the cyclization states of gRNA.

### Specificity of the N protein for melting and annealing activity

Another way to infer the specific functions of N and its role in gRNA replication and template switching is through its RNA chaperone activity, which is defined by N's ability to facilitate the interconversion of gRNA conformational states within the thermodynamic energy landscape (97). The NTD can promote either melting or annealing of duplex RNA. In Table 2, we compiled the available experimental data describing the target RNA/DNA sequences and the reported activities.

**Table 2:** Compilation of reported experimental annealing and melting activities of N protein

Nucleic acid origin	Type of activity	Sequence	Citation
TRS-cTRS duplex TGEV and SARS-CoV (RNA)	Annealing (<1 μM N)	5′-UCG**AA**CU**AAA**CG**AAA**U-3′	Zuñiga *et al.* (98)
		3′-UGCUUGAUUUGCUCUA-5′	
TRS hairpin TGEV and SARS-CoV (RNA)	Annealing (<1 μM N)	5′-UCG**AA**CU**AAA**CG**AAA**U-3′	Zuñiga *et al.* (98)
Oligos B and C duplex DNA	Annealing (<1 μM N)	5′-TTATT**AA**CCCTCACT**AAA**-3′	Zuñiga *et al.* (98)
		3′-AATAATTGGGAGTGATTT…(ss)…-5′	
TRS-cTRS duplex TGEV (RNA)	Annealing (<1 μM)	5′-UCGAACU**AAA**CG**AAA**U-3′	Zuñiga *et al.* (10)
		3′-UGCUUGAUUUGCUCUA-5′	
Oligos B and C duplex DNA	Annealing (<1 μM)	5′-TTATT**AA**CCCTCACT**AAA**-3′	Zuñiga *et al.* (10)
		3′-AATAATTGGGAGTGATTT…(ss)…-5′	
TRS-cTRS duplex MHV (RNA)	Melting	5′-**AA**UCU**AAA**CU-3′	Grossoehme *et al.* (55)
		3′-UUAGAUUUGA-5′	
SL3 SARS-CoV (RNA)	Melting	5′-GUUCUCU**AAA**CGAAC-3′	Grossoehme *et al.* (55)
TRS-cTRS duplex MHV (RNA)	Melting	5′-**AA**UCU**AAA**CU-3′	Keane *et al.* (50)
		3′-UUAGAUUUGA-5′	
TRS-cTRS duplex SARS-CoV-2 (DNA)	Melting	5′-TCT**AAA**CCGCG-3′	Caruso *et al.* (21)
		3′-AGATTTGGCGC-5′	
TRS-cTRS duplex SARS-CoV-2 (RNA)	Melting	5′-UCU**AAA**CCGCG-3′	Caruso *et al.* (21)
		3′-AGAUUUGGCGC-5′	
Ext hairpin of SL4 SARS-CoV-2 (RNA)	Melting	5′-GGAU**AA**UU**AA**U**AA**CU**AA**UUACU-3′	Korn *et al.* (48)
SL3 SARS-CoV-2 (RNA)	Melting	5′-…GUUCUCU**AAA**CGAAC…-3′	Korn *et al.* (48)
SL2 SARS-CoV-2 (RNA)	No activity	5′-…GAUUCCUUGUAGAUC…-3′	Korn *et al.* (48)
5′-OhS22D21 duplex DNA overhang of 21 nt	Melting activity (>1 μM)	5′-TAC**AA**TGT**AAA**T**AAAA**CATCG-3′	Zhang *et al.* (62)
		3′-ATGTTACATTTATTTTGTAGC…(ss)…-5′	
5′-OhS22D21 duplex DNA overhang of 21 nt	Annealing (0.01–1 μM)	5′-TAC**AA**TGT**AAA**T**AAAA**CATCG-3′	Zhang *et al.* (62)
		3′-ATGTTACATTTATTTTGTAGC…(ss)…-5′	
3′-OhS22D21 duplex DNA overhang of 21 nt	No activity (3 μM)	5′-ATGTTACATTTATTTTGTAGC-3′	Zhang *et al.* (62)
		3′-TACAATGTAAATAAAACATCG…(ss)…-5′	
5′-OhS4D20 duplex DNA overhang of 4 nt	No activity (3 μM)	5′-CATCTGCCGG**AA**TGACAT**AA**-3′	Zhang *et al.* (62)
		3′-GTAGACGGCCTTACTGTATT…(ss…-3′	
DS32 duplex DNA	No activity	5′-CATCTGCCGG**AA**TGACAT**AA**CATTCTTCGATA-3′	Zhang *et al.* (62)
		3′-ATAGCTTCTTACAATACAGTAAGGCCGTCTAC-5′	
Nonspecific duplex RNA	No activity	5′-CACUGACCGCG-3′	Personal communication
		3′-GUGACUGGCGC-5′	

**Figure 4:**
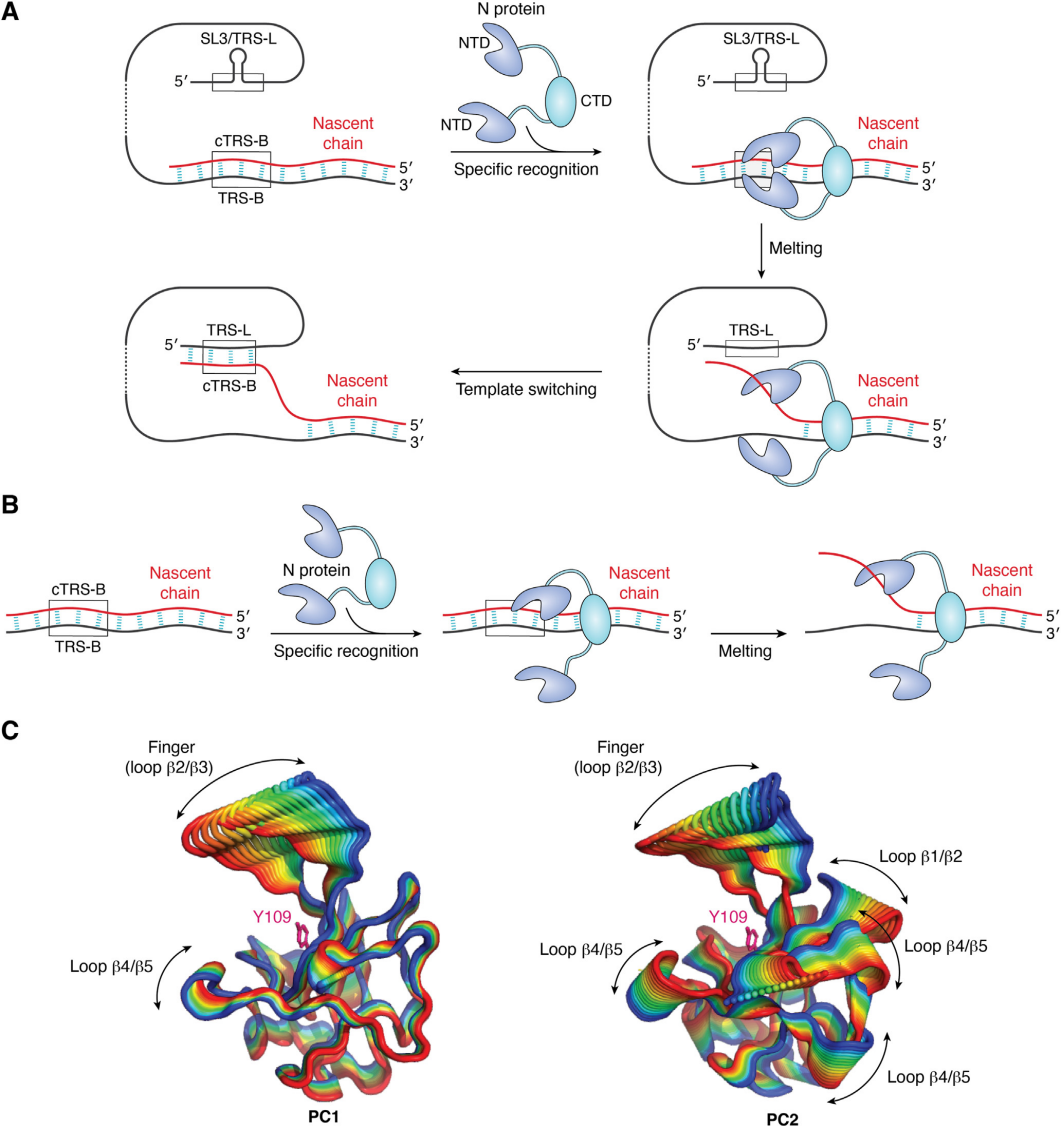
**Models of the double-stranded RNA (dsRNA) melting mechanism promoted by the N protein.***A*, the sandwich model was proposed by Grossoehme and colleagues (55).The polymerase synthesizes the negative-sense nascent chain (*red*). The dimeric form of the N protein scans the duplex RNA and recognizes TRS-B/cTRS-B (dsTRS), making a sandwich: one N-terminal domain (NTD) subunit binds to TRS-B, and the other binds to cTRS-B. dsTRS-B is destabilized, leading to the dissociation (melting) of the dsTRS. Each strand (TRS-B, cTRS-B) becomes associated with each NTD subunit. The melting of dsTRS promoted by N facilitates template switching, as described in Figure 2. Note that for the annealing of cTRS-B with TRS-L, the unwinding of SL3, which contains the TRS, is necessary. *B*, in the second model, only one NTD recognizes and melts the dsTRS. The possibility of two subunits exerting independent activities increases the efficiency of the mechanism. *C*, NTD motions filtered from principal component analysis of 25 runs of MD simulation of the SARS-CoV-2 NTD binding a duplex TRS-L and cTRS-L (dsTRS-L) (99). The colored gradient represents the direction of the motions from *blue* to *red*. PC1 converges to a tweezer-like motion, mainly through the finger region. PC2 shows a twist-like motion of the palm, which involves the long β4/β5 loop, β1/β2 loop, and finger. As a reference, the figure shows that Y109 is located at the center of the binding site.

The first description of N chaperone activity was provided by Zuñiga *et al.* (98). They demonstrated that full-length TGEV N enhances hammerhead ribozyme self-cleavage, a classical *in vitro* method used to confirm chaperone activity. They also used electrophoretic mobility shift assays (EMSAs) to show that TGEV and SARS-CoV full-length N can anneal the single-strand TRS and cTRS into dsTRS, as well as anneal the single-strand TRS into a hairpin, forming the structure of SL3 (Table 2 and Fig. 4*A*). Additionally, N exhibits annealing activity toward complementary strands of non-TRS DNA oligonucleotides (Table 2).

In 2010, the same authors provided the most direct evidence of N's role in template switching. Using an *in vitro* retrovirus-derived heterologous system, they demonstrated that the TGEV N can function as an RNA chaperone, enhancing the template switch (10). Interestingly, this annealing activity is not limited to the full-length N. Constructs containing N-arm-NTD, N-arm-NTD-linker, or even just the linker alone can also promote the annealing of dsTRS RNA and a duplex DNA sequence (Table 2). This annealing activity is consistently observed at low concentrations of N, typically in the submicromolar range, and at nearly equimolar concentrations of ssRNA.

Grossoehme *et al.* (55) were the first to demonstrate that at higher concentrations, N can promote the melting of dsTRS. They employed fluorescence resonance energy transfer (FRET) *in vitro* assays to measure the unwinding of duplex RNA or the linearization of RNA hairpins. Their findings revealed that the MHV and SARS-CoV N proteins facilitated the melting of dsTRS-L into TRS-L and cTRS-L, as well as the melting of TRS-L (SL3 hairpin) into its unannealed conformation (Table 2). The presence of the SR region significantly enhanced this melting activity. Subsequent studies confirmed the melting activity of dsTRS for both RNA and DNA in SARS-CoV-2 (21). As mentioned earlier, Korn *et al.* (48) investigated the impact of the SARS-CoV-2 NTD on structural elements of the 5′UTR. Using the disappearance of ^1^H-NMR imino resonances as a probe for melting activity, they demonstrated that N melted SL3 (TRS-L) but not SL2 when it acted on an RNA oligonucleotide containing both SL2 and SL3. N also melted the hairpin Ext (an extension of SL4, Table 2). This melting activity was consistently observed at higher concentrations of N, typically with an excess of N relative to dsRNA.

On the basis of the available data, it can be inferred that the NTD shows specificity for both melting and annealing toward sequences containing multiple adenines (A). The most common motif is a triple-A (AAA) in the positive-sense strand, which is present in most TRSs. NTD also exhibits activity toward motifs with multiple double-A (AA) repeats in the positive-sense strand, such as the Ext hairpin sequence (UAAUUAAUAACUAAU). To date, the only experimentally confirmed RNA/DNA sequences in which the NTD is inactive do not contain triple-A or multiple AA motifs, including nonspecific RNA (CACUGACCGCG) and SL2 (GAUUCCUUGUAGAUC) (Table 2).

Zhang *et al.* (62) investigated various DNA sequences and observed either melting or annealing activity, suggesting that the unwinding polarity depends on the presence of a 5′ DNA overhang. We offer an alternative explanation, suggesting that this activity is influenced by the presence of AAAs or multiple AA motifs on the positive-sense strand, such as in the 5′-OhS22D21 sequence (Table 2). The authors named the oligonucleotides *via* the following pattern: polarity (5′ or 3′), overhang (Oh), number of nucleotides in the single strand (S22), and number of nucleotides in the double strand (D21). Notably, the NTD remains inactive when the AAA motif is located on the negative-sense strand, as observed in 3′-OhS22D21 (Table 2). Additionally, the NTD shows no activity toward 5′-OhS4D20 and DS32 dsDNA, which contain two AA sequences (…GAATGACATAA). This finding indicates that the mere presence of multiple AA motifs may not be sufficient for activity; the spacing between these motifs could also be crucial. Given the limited information currently available, we can cautiously conclude that the NTD is specific to AAAs and multiple AA sequences. However, further research is needed to define the active motifs precisely.

Two models have been proposed for the melting mechanism of the NTD. The first model suggests the formation of a ternary complex where the dsTRS is sandwiched by two NTDs (55) (Fig. 4*A*). According to this model, the N protein scans the negative-sense nascent chain until it encounters the specific TRS CS. The authors considered the dimeric nature of the protein. Each NTD binds specifically to the dsTRS, leading to its dissociation. Each NTD subunit subsequently binds to single-stranded TRS-B and cTRS-B, which facilitates the pairing of cTRS-B with TRS-L and promotes template switching. Note that SL3 must be unwound to pair with cTRS-B (Fig. 4*A*).

The second proposed model involves only a single NTD subunit to facilitate the unwinding of the dsTRS (Fig. 4*B*). This model, proposed by our group in 2021 (99), is based on the destabilization of dsTRS observed at the binding cleft of the SARS-CoV-2 NTD through molecular dynamics (MD) simulations. In this work, we run 25 replicas of 100 ns MD simulations for the dsTRS free and in the presence of the NTD. We also run the MD simulation for a nonspecific dsRNA (dsNS). The results showed that for the free dsTRS and dsNS and for the dsNS bound to the NTD, the dsRNA structure was intact throughout all MD simulations, with all Watson–Crick hydrogen bonds present. Remarkably, for the dsTRS bound to the NTD, in 4 out of 25 replicates, we observed dsRNA destabilization, resembling the melting of the dsTRS at the NTD RNA binding cleft. The Watson–Crick hydrogen bonds of the dsTRS are replaced by hydrogen bonds between the nitrogenous bases and the protein. This destabilization was not observed with non-TRS dsRNA sequences or free dsRNAs. The model is further supported by simulations of the kinetics of dsRNA melting, which replicate experimental melting curves obtained from FRET-based dsRNA dissociation assays (21, 50, 55, 99).

The main driving force behind the dissociation of dsTRS at the binding cleft is attributed to the dynamics of the NTD (99). Principal component analysis (PCA) of the MD simulations revealed a tweezer-like motion between the finger and the β4/β5 large loop, as observed in the first principal component (PC1, Fig. 4*C*). Another dynamic feature is the twist of the β-sheet (palm), which is observed in the second principal component (PC2, Fig. 4*C*).

Neither of the proposed models is fully experimentally validated, but there are some considerations to note. The sandwich model assumes specific recognition of both TRS-B and cTRS-B. This is unlikely since the specific motif is generally established to be in the positive-sense strand (Table 2). Additionally, the kinetic simulation of dsRNA-melting activity did not yield melting curves consistent with experimental observations (99). The second model suggests that each NTD in the dimeric N protein functions independently (Fig. 4*B*), potentially offering greater efficiency than the sandwich model does. In this model, one NTD could unwind dsTRS-B, whereas the other NTD could facilitate the unwinding of SL3, as observed experimentally (48, 55). The two NTD arms could bind to different but spatially close RNA sites in the viral genome. This binding could promote bridging between the segments, similar to what Lo *et al.* reported for bovine betacoronavirus (100).

### Viral genome structure

As mentioned before, template switching results from polymerase jumping, which occurs at low frequencies independently of the TRS. N facilitates this process, making template switching more efficient. Therefore, understanding the conformational dynamics of the gRNA is essential. Next, we review the genome architecture of SARS-CoV-2 gRNA as described in the literature.

In addition to carrying genetic information, RNA molecules can also mediate biological processes because of their ability to fold into secondary and tertiary structures. Structure-specific chemical modification techniques, coupled with next-generation sequencing, enable experimental probing of RNA secondary structures in large RNA segments, such as viral gRNA, providing single-nucleotide resolution structural data.

These methods rely on the chemical modification of the gRNA. Dimethyl sulfate (DMS) modifies unpaired adenines and cytosines, with methylation profiles of m^1^A and m^3^C (101). Selective 2′-hydroxyl acylation, analyzed by primer extension (SHAPE), promotes the formation of covalent 2′-O-adducts at RNA nucleotides that are conformationally flexible, mapping the presence of loops, bulges, and other unpaired nucleotides (102). Both tools can be coupled with a mutational profiling (MaP) approach, which uses reverse transcriptase to encode the chemically modified bases as mutations in the cDNA. These cDNAs are then subjected to massively parallel sequencing, and the profiles are reconstructed from mutation frequencies derived from all sequencing reads (101, 102). These methods have been applied to determine the secondary structure of SARS-CoV-2 gRNA (94, 103, 104, 105, 106, 107) and the 5′ UTR, 3′ UTR, and frame-shifting element pseudoknot, as confirmed by NMR (75). Many structural elements are conserved among coronaviruses (105). Another important feature is the plasticity of some structural elements, such as the 5′ UTR SL3 of SARS-CoV-2, which adopts alternate conformations (104, 108).

**Figure 5:**
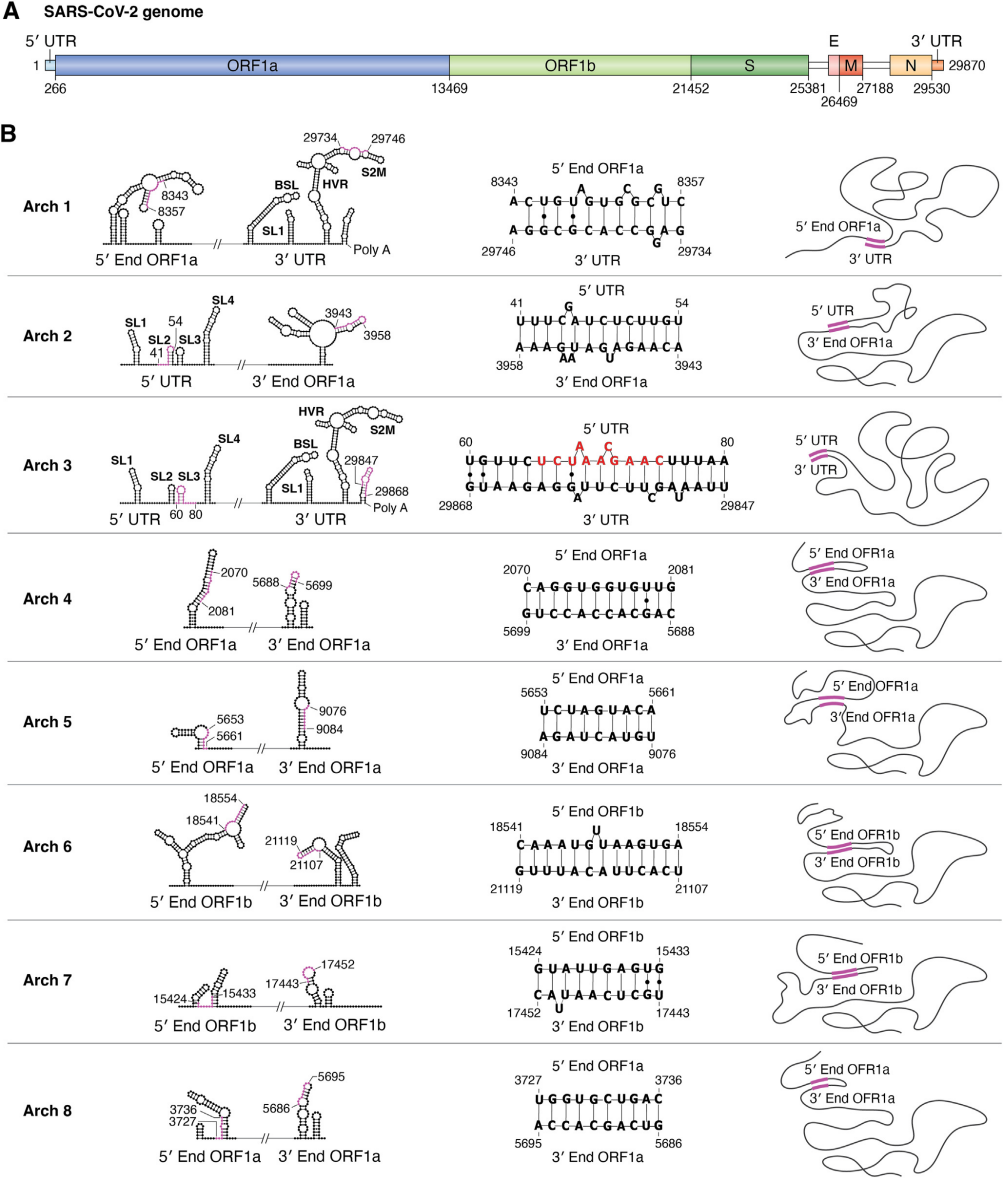
**SARS-CoV-2 genome architectures experimentally reported by Ziv *et al.*** (95)**.***A*, representation of the SARS-CoV-2 gRNA used as a guide. The size of each region is scaled to the size of the genome. *B*, Ziv architectures described in a previous paper (95). The first column shows the name of each architecture, numbered from one to eight. The second column shows the secondary structure of each interacting region calculated by RNAfold WebServer (113). The complementary regions involved in the interactions are colored in *magenta*. The third column shows the RNA sequences and their positions at the gRNA. Noncanonical base pairs are represented as *black dots*. The sequence in *red* for Arch 3 is TRS-L. The fourth column is a free drawing of the interaction to help visualize the effect of each gRNA cyclization.

The next important aspect is the tertiary architecture that the gRNA can assume. Elucidating the structural and dynamic landscape of a viral genome is challenging but essential for fully understanding replication and transcriptional regulation. Ziv *et al.* (109) developed a methodology named crosslinking of matched RNAs and deep sequencing (COMRADES), which retrieves *in vivo* information on short- and long-range RNA–RNA interactions. In SARS-CoV-2, distinct genome architectures of higher-order conformations were reported to be sustained through base pairing (Fig. 5) (95). They described eight architectures revealing the complex conformational dynamics of the gRNA. Each architecture (Archs 1–8, Fig. 5*B*) describes the long-range base pairing of the gRNA that may coexist in different discrete conformational states, offering a means to rationalize different patterns of template switching (28).

Arch 3 defines long-range base pairing between the 5′ and 3′ UTRs, creating a cyclization pattern that places the unwound SL3 close to the 3′ polyA, supporting the template switch (28, 95). Both regions involved in the base pairing of genome cyclization have structures determined by NMR spectroscopy (75) and show evidence of dynamics with alternate conformations. Notably, the positive-sense strand contains the TRS triple-A motif, suggesting that the N protein may play a role in these cyclization dynamics, either through its annealing or melting activities.

Arch 1 and Arch 2 connect ORF1a with the 3′ and 5′ UTRs, respectively. They are likely important for encompassing the template switch, as they may indirectly link the 5′ and 3′ UTRs. Archs 4 to 8 are responsible for the cyclization of ORF1a (Archs 4, 5, and 8) and ORF1b (Archs 6 and 7). These long-range interactions may also contribute to template switching. Interestingly, Arch 6 contains the triple-A motif and could be a site for N chaperone activity.

Despite the success of COMRADES in describing long-range RNA–RNA interactions, Zhang *et al.* (110) detected the direct interaction of TRS-L with several TRS-Bs *via* the sequencing of psoralen crosslinked, ligated, and selected hybrids (SPLASH). This provides the first direct experimental evidence of the long-range interactions required for template switching. As discussed earlier, the involvement of the N protein in interactions involving TRS-L is well established by both *in vitro* and *in vivo* experiments (21, 50, 55, 85, 86, 87).

These data suggest that genome cyclization and template switching can be regulated by dynamic contributions. This is supported by experimental measurements from NMR spectroscopy for the 5′ UTR SL4 (106) and DMS-MaPseq structural models of the 3′ UTR of SARS-CoV-2, which show at least two different conformations in the nucleotide window from 29,546 to 29,767 (111). The conformational flexibility and structural maintenance of the genome, including pseudoknots and base-pairing interactions, are crucial for viral infectivity. According to Huston and colleagues (94), well-folded regions in ORF1a of SARS-CoV-2 (nucleotides 7717–8230 and 10,798–11,039) may play regulatory roles in the virus life cycle. They demonstrated that disruption of these secondary structures results in decreased viral growth (94).

## Conclusion

Overall, we have provided experimental evidence that the conformational dynamics of the gRNA are crucial for template switching and, consequently, for discontinuous transcription. This dynamic behavior is also important for genome replication and nucleocapsid packing. The N protein plays a key role in these critical events of the viral cycle, increasing its efficiency through its RNA chaperone activity by promoting melting and annealing at specific duplex RNA regions and hairpins.

N stands out as a multifunctional protein that is promiscuous for the recognition of RNA while performing specific functions centered on the NTD. We reviewed the structural basis for this specificity, which is directly linked to its regulatory roles. These activities include dsRNA annealing and melting, facilitating template switching, and enabling discontinuous transcription. The N protein's RNA chaperone activity and its capacity to promote LLPS are central to its multiple functions in the viral cycle. Additionally, N activity is finely regulated through posttranslational modifications. While phosphorylation is the most studied process, N also undergoes methylation, acetylation, and glycosylation, which are still not well understood.

To elucidate the role of N in replication, discontinuous transcription, and genome reorganization, we believe that future experiments should address the following aspects:

(1) Pinpoint the specific interaction regions of N with the gRNA at different phases of the viral cycle.
(2) Better characterization of the RNA-specific motifs of the N protein involved in melting and annealing activity is needed. This may help elucidate the impact of N on the conformational dynamics of the gRNA, given that N is the most abundant interaction partner with the gRNA.
(3) New biophysical experiments should be conducted to further understand the N melting/annealing mechanism.
(4) Most studies on melting and annealing activity have used small oligonucleotides. We suggest that binding affinity to the gRNA is likely highly dependent on its conformation. Future studies with larger RNA segments from the genome may provide insight into the role of local RNA conformation in chaperone activity.
(5) Establishing how LLPS modulates N chaperone activity. Presumably, LLPS plays an important role in local RNA concentration and, consequently, chaperone activity.
(6) New studies are needed to provide direct experimental evidence of the involvement of N in the template switch mechanism.

## Data availability

All data are included within the article.

## Supporting information

This article does not contain supporting information.

## Conflict of interest

The authors declare that they have no conflicts of interest with the contents of this article.
